# Astrocytes Decreased the Sensitivity of Glioblastoma Cells to Temozolomide and Bay 11-7082

**DOI:** 10.3390/ijms21197154

**Published:** 2020-09-28

**Authors:** Sadaf E. Pustchi, Naze G. Avci, Yasemin M. Akay, Metin Akay

**Affiliations:** Department of Biomedical Engineering, University of Houston, Houston, TX 77204, USA; sebrahi9@uh.edu (S.E.P.); ngavci@uh.edu (N.G.A.); ymakay@uh.edu (Y.M.A.)

**Keywords:** 3D co-culture, glioblastoma, astrocytes, tumor microenvironment, PEGDA

## Abstract

Glioblastoma multiforme (GBM) is the most common malignant type of astrocytic tumors. GBM patients have a poor prognosis with a median survival of approximately 15 months despite the “Stupp” Regimen and high tumor recurrence due to the tumor resistance to chemotherapy. In this study, we co-cultured GBM cells with human astrocytes in three-dimensional (3D) poly(ethylene glycol) dimethyl acrylate (PEGDA) microwells to mimic the tumor microenvironment. We treated 3D co- and mono-cultured cells with Temozolomide (TMZ) and the nuclear factor-κB (NF-κB) inhibitor Bay 11-7082 and investigated the combined effect of the drugs. We assessed the expressions of glial fibrillary acidic protein (GFAP) and vimentin that play a role in the tumor malignancy and activation of the astrocytes as well as Notch-1 and survivin that play a role in GBM malignancy after the drug treatment to understand how astrocytes induced GBM drug response. Our results showed that in the co-culture, astrocytes increased GBM survival and resistance after combined drug treatment compared to mono-cultures. These data restated the importance of 3D cell culture to mimic the tumor microenvironment for drug screening.

## 1. Introduction

Glioblastoma multiforme (GBM), a grade IV tumor based on the WHO classification, is the most common malignant type of astrocytic tumors [1]. GBM patients usually have a median survival of approximately 15 months with the “Stupp” Regimen, consisting of 6 weeks of radiation with daily Temozolomide (TMZ) followed by 6 cycles of TMZ [2,3]. However, despite the modern therapies, GBM patients still have poor prognosis and high tumor recurrence due to the tumor resistance to chemotherapy [4,5]. TMZ, a DNA alkylating agent, is currently the most widely used and effective chemotherapeutic drug for GBM [6]. One of the important mechanisms of TMZ resistance is the increased activity of O_6_-methylguanine-DNA methyltransferase (MGMT), which can repair the TMZ-induced guanine damage in DNA lesions [7]. However, the underlying molecular mechanism of TMZ resistance is more complex than its dependence on MGMT expression [8]. Another mechanism that is associated with TMZ resistance in GBM is the excessive activation of Nuclear Factor-κB (NF-κB) [9]. It was shown that NF-κB inhibitor Bay 11-7082 suppressed MGMT and promoted TMZ-induced cytotoxicity and apoptosis in U251 GBM cells [8]. This suggests that the combination of TMZ and Bay 11-7082 drugs have a combinatorial effect of inhibiting GBM growth [10].

The tumor microenvironment has a crucial role in cancer development, progression, and response to treatment [5]. The GBM microenvironment does not only contain tumor cells but also a mixture of diverse stromal cell types. Those include endothelial cells (EC), which constitute blood vessels, microglia contributing to resistance of antiangiogenic therapy, and an extra-cellular matrix consisting of a mix of collagen, proteoglycans, fibers, and astrocytes [11,12,13,14]. Astrocytes comprise ~50% of all brain cells, support the neural network, promote cell–cell communications, and participate in the formation, development, and maintenance of neural circuits in the central nervous system (CNS) [13,15,16]. In malignant diseases of the CNS, reactive astrocytes can impact the course of the disease. Tumor-associated astrocytes promote tumor malignancy by causing cell proliferation and migration [16] and contribute to anti-inflammatory responses [17]. Reactive astrocytes are characterized by the upregulation of two cytoplasmic intermediate filament proteins (IF), glial fibrillary acidic protein (GFAP) and vimentin [15]. GFAP is the major protein involved in cell communication and provides mechanical support to cells and the functioning of the blood–brain barrier [18]. Vimentin is the major cytoskeletal component present in immature glia and a biomarker of cellular epithelial to mesenchymal transition (EMT). It is known to play a crucial role in the maintenance of cellular integrity and regulating GBM cell invasion and metastatic tumor spread [19,20]. Its expression has been reported in most of the glioma cell lines, such as U-118MG, U-373MG, and U-87MG [19]. Although vimentin has been used as a biomarker, its role in GBM has not been explored in detail. Notch-1 and survivin are associated with GBM apoptosis and proliferation in tumor microenvironment [21,22]. The Notch-1 signaling pathway regulates important biological processes such as cell proliferation, apoptosis, migration, self-renewal, and differentiation [23,24]. Notch-1 overexpression correlates with a high grade glioma and a poor prognosis, suggesting that upregulated Notch-1 signaling promotes a more undifferentiated and aggressive tumor phenotype [21,25]. Survivin is a member of the inhibitor-of-apoptosis (IAP) family and its overexpression suppresses cell death [24]. Its expression has been associated with enhancing malignant potential of gliomas [25]. Survivin expression has been reported as a risk factor for resistance to radiation and chemotherapy in GBM [26].

Traditionally, two-dimensional (2D) models are used to study drug treatments, wherein tumor cells are cultured as a monolayer and do not closely mimic the tumor physiological microenvironment [27]. Cancer cells grown on a flat 2D surface lose crucial signaling pathways which define natural responses of the cells’ tumor growth, metabolism, and differentiation [28]. Moreover, significant differences have been shown in cell behavior, gene expression, and drug response when cells were cultured in three-dimensional (3D) compared with 2D monolayer culture [29,30]. In 2D cultures, cells promoting cell-to-cell and cell-to-matrix interactions and displaying a hypoxic core that is more resistant to chemotherapy, immunotherapy, and radiation therapy cannot be observed thoroughly [29]. Therefore, in drug screening, 2D cultures may not be the best representation of the in vivo tumor microenvironment. A simulation of the tumor microenvironment in a 3D culture model would give more precise information in addressing the challenges of drug response and resistance.

Several hydrogel microfabrication technologies have been used to develop biologically relevant 3D scaffolds, including photolithography, 3D bioprinting, and microfluidics [31,32,33]. Photolithography is one of the prominent techniques in 3D scaffold fabrication due to the simple and cost-efficient preparation process [31]. Photomask-based photolithography is a conventional type of photolithography to build a 3D scaffold which does not require a silicon mold or PDMS (polydimethylsiloxane) stamp [32]. This method has the advantages of cost-efficient preparation process, high reproducibility, and convenient control over the size and shape of the micropattern [33]. Hydrogels are one of the most common tissue engineering scaffolds due to their ability to maintain a precise 3D structure, contribute to the mechanical support of the cells, and simulate the extracellular matrix [34]. Poly(ethylene glycol) dimethyl acrylate (PEGDA) is used to fabricate 3D scaffolds with photolithography for its biocompatibility, ease of crosslinking to create a hydrogel, and finely tunable bioactivity [34].

In our lab, we used 3D GBM spheroid systems for several studies, including PEGDA hydrogel microwells, in order to understand the effect of the tumor microenvironment on GBM growth [33,35]. We also investigated the interaction of 3D GBM spheroids with endothelial cells [36]. In this study, we explored the interaction of 3D GBM spheroids with astrocytes before and after drug treatment. GBM cells were co-cultured with human astrocytes in PEGDA microwells. Then, they were treated with TMZ and/or NF-κB inhibitor (Bay 11-7082) to understand the effect of astrocytes in GBM growth and drug response. Furthermore, using real-time PCR (qPCR) and Western blot analysis, the expressions of proteins that play role in the activation of astrocytes, such GFAP and vimentin, as well as GBM malignancy, such as Notch-1 and survivin, were investigated in the drug-treated LN229–astrocyte co-culture and compared with the mono-cultures. Our data indicated that the presence of astrocytes increased cell viability and the expression of the proteins in the co-culture after the drug treatment compared with the mono-cultures. These results showed the importance of the tumor microenvironment in the GBM growth, proliferation, and drug resistance.

## 2. Results

### 2.1. Microwell Preparation

**Figure 1:**
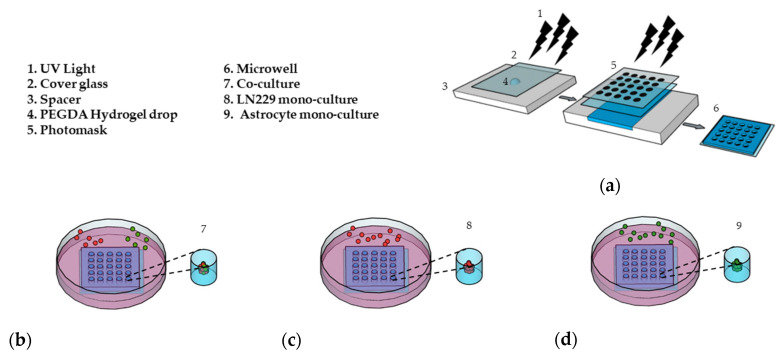
Preparation of the 3D cell culture in the (PEGDA) microwells. (**a**) Schematic of the PEGDA microwells’ fabrication using a round pattern photomask (400 µm in diameter). (**b**) LN229 cells stained with a red cell tracker and astrocytes stained with a green cell tracker were added into the microwells and co-cultured with a ratio of 1:1 (LN229:astrocytes) for 7 days. (**c**) LN229 cells were mono-cultured into microwells for 7 days. (**d**) Astrocytes cells were mono-cultured into microwells for 7 days.

PEGDA microwells were fabricated as previously described [33,34], as shown in Figure 1a. Photoresponsive hydrogels are composed of a polymeric network and photoreactive moiety, which capture the optical signal and transfer it to the functional part of the polymers [37]. The hydrogel solution is exposed to UV light through a patterned photomask in the presence of a photoinitiator. Transparent regions of the photomask cause photoreaction, which leads to crosslinking in the polymer and forms hydrogel microwells [38]. The PEGDA microwell stability was due to 3-(Trimethoxysilyl) propyl methacrylate (TMSPMA) treatment on the cover glass surface, which provides covalent bonding between the glass surface and PEGDA hydrogel [39]. Fabricated microwells were able to maintain their stability without detaching from the cover glass for a total of 14 days, as we previously showed [33].

### 2.2. Cell Culture and Drug Application

For the first 7 days, we co-cultured LN229 cells and astrocytes with a ratio of 1:1 (Figure 1b), mono-cultured LN229 (Figure 1c), and astrocytes (Figure 1d) in the microwells to generate 3D spheroids. In this study, we used 600 µM of TMZ and 10 µM of Bay 11-7082 concentration on day 7 and cultured 3D spheroids for an additional 7 days since astrocytes have been found to be more TMZ resistant [40,41].

**Figure 2:**
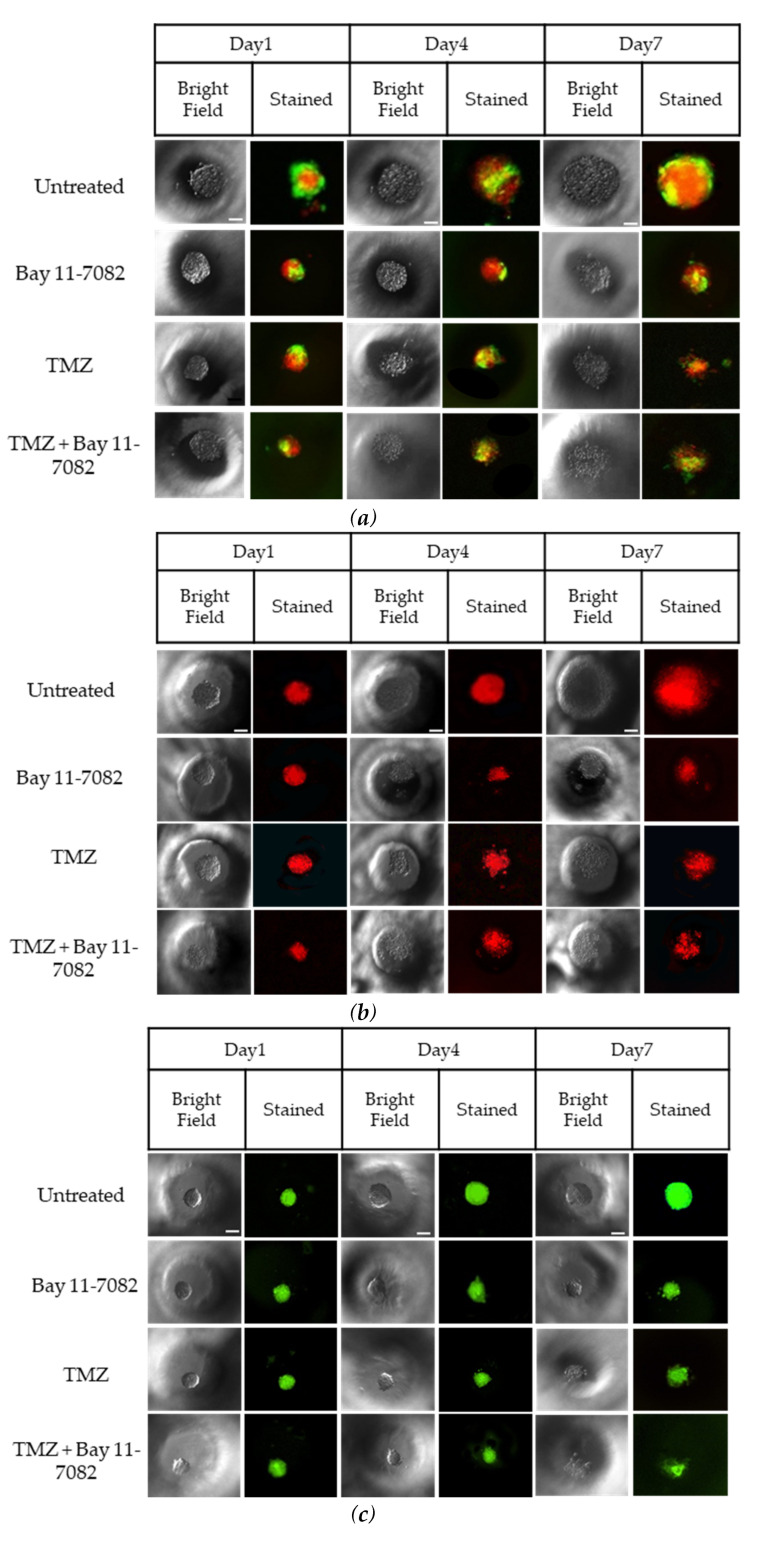

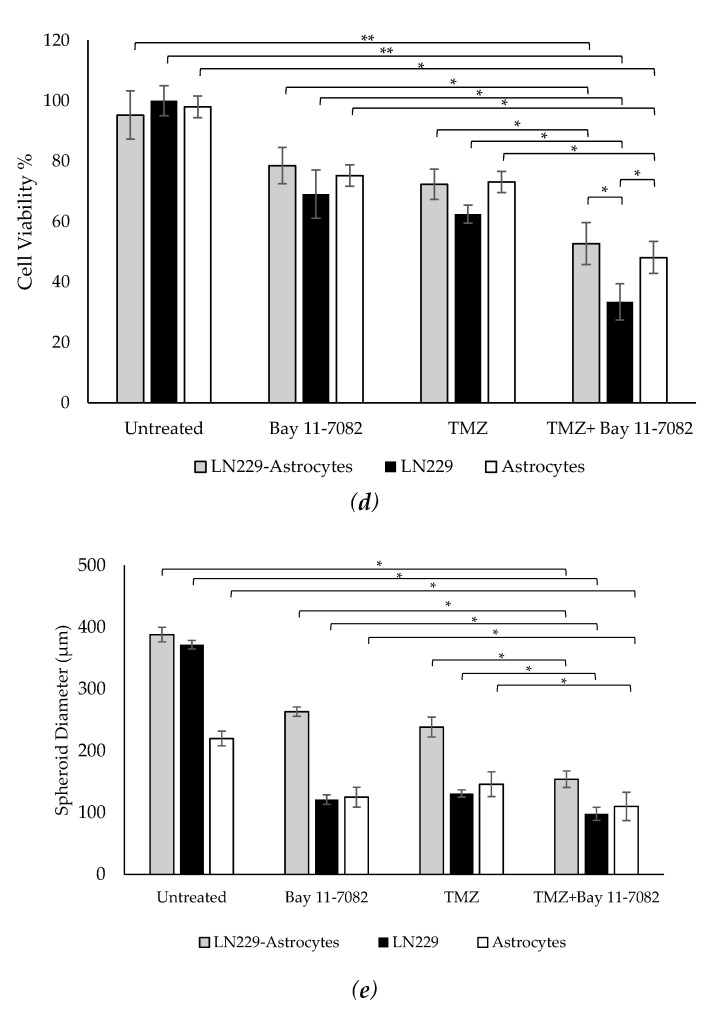
Representative images of co-cultured and mono-cultured cells in the PEGDA microwells (400 µm) after 7 days of drug administrations: untreated, Bay 11-7082, Temozolomide (TMZ), and TMZ–Bay 11-7082. Effects of the drug treatment on both co-culture and mono-culture were visualized by the disaggregation of the dead cells on day 7. LN229 and astrocytes cells were stained with cell tracker red and cell tracker green, respectively. (**a**) LN229–astrocytes (1:1) co-culture. (**b**) LN229 mono-culture. (**c**) Astrocytes mono-culture. (**d**) Viability of co-cultured and mono-cultured cells. (**e**) Diameter of co-cultured and mono-cultured spheroids (µm). * indicates *p* < 0.05, ** indicates *p* < 0.01. X20 objective. Scale bars, 100 µm.

The correlation between the cell seeding concentration and the size of the microwells has been previously optimized in our lab [33]. To evaluate the effect of astrocytes on GBM drug sensitivity, LN229 cells and astrocytes were co-cultured in the microwells with a concentration of 0.2 × 10^6^ cells/mL using a ratio of 1:1. In order to distinguish LN229 cells and astrocytes in the co-culture, LN299 cells were stained with a red cell tracker and astrocytes were stained with a green cell tracker. LN229 cells and astrocytes were mono-cultured separately with a concentration of 0.2 × 10^6^ cells/mL in the microwells. Using fluorescence microscopy, we observed that untreated groups in the LN229–astrocytes co-culture (Figure 2a), LN229 (Figure 2b), and astrocytes (Figure 2c) mono-cultures formed stable 3D spheroids after 7 days in the microwells.

Trypan blue cell viability assay was performed after the drug introduction to both co-cultured and mono-cultured cells on day 7 to understand the combined drug effect on the 3D spheroids. As shown in (Figure 2d), the drug treatment resulted in a decrease in the cell viability of co-cultured cells. On day 7, the cell viability data of the co-cultured cells were 87.33 ± 7.9% for the untreated group, 72 ± 5.4% after Bay 11-7082, 66.33 ± 5.7% after TMZ, and 48.28 ± 4.8% after TMZ–Bay 11-7082 treatment. The cell viability after TMZ–Bay 11-7082 treatment was significantly lower compared with the untreated group (*p* < 0.01). In addition, the cell viability significantly decreased after the combined drug treatment compared with both single-drug treatment groups (*p* < 0.05). The cell viability in the LN229 mono-culture after combined drug treatment was 30.66 ± 5.2% and was significantly lower than the untreated group (91.66 ± 4.2%) (*p* < 0.01). LN229 mono-cultured cells treated with Bay 11-7082 alone and TMZ alone showed 63.33 ± 3.8% and 57.43 ± 4.5% cell viability, respectively, and they were both significantly higher than combined drug treatment in mono-culture on day 7 (*p* < 0.05). The cell viability in astrocytes mono-culture after combined drug treatment was 48.09 ± 5.29% and was significantly lower than the untreated group 98.0 ± 3.62% (*p* < 0.05). Astrocyte mono-culture treated with Bay 11-7082 alone and TMZ alone showed 75.23 ± 3.51% and 73.14 ± 3.62% cell viability, respectively, and they were both significantly higher than combined drug treatment in mono-culture on day 7 (*p* < 0.05). Our results also showed that the cell viability in LN229 significantly decreased compared with the co-culture and astrocyte mono-culture treated with combined drugs (*p* < 0.05). The cell viability data showed the combined treatment was more effective than using a single drug treatment, and it can be presumed that the presence of astrocytes in the co-culture significantly decreased drug effectiveness compared to mono-culture.

The size of the spheroids in the co-culture and mono-cultures on day 7 after drug administration was measured using ImageJ (Figure 2e). The results showed that the size of the spheroids in the co-culture was 388 µm in the untreated group, 263.33 µm after Bay 11-7082, 238.3 µm after TMZ, and 154 µm after TMZ–Bay 11-7082 treatments. There was a significant decrease in the spheroid size of the co-culture after combined drug treatment compared with the untreated group (*p* < 0.05). Mono-culture LN229 spheroid size was 371.66 µm in the untreated group, 121 µm after Bay 11-7082, 134 µm after TMZ, 98.1 µm after TMZ–Bay 11-7082 treatments, and there was a significant decrease in the spheroid size of the mono-culture after combined drug treatment compared with the untreated group (*p* < 0.05). Spheroid sizes in the astrocytes mono-culture were 220 µm in the untreated group, 125 µm after Bay 11-7082, 146 µm after TMZ, and 110 µm after TMZ–Bay 11-7082 treatments. The decrease in the spheroid size of the astrocyte mono-culture after TMZ–Bay 11-7082 treatment was significant compared to the respective untreated group (*p* < 0.05).

**Figure 3:**
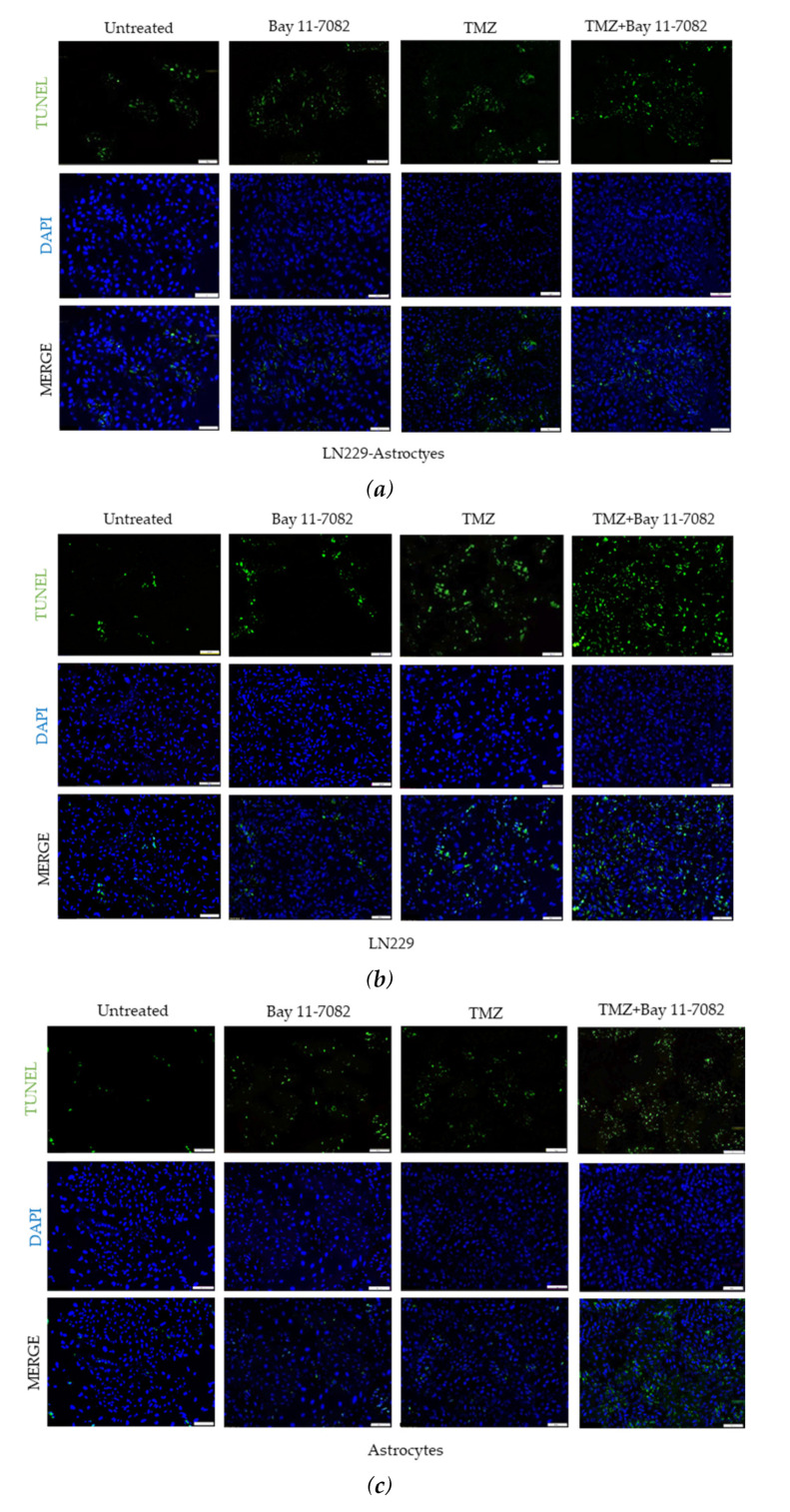

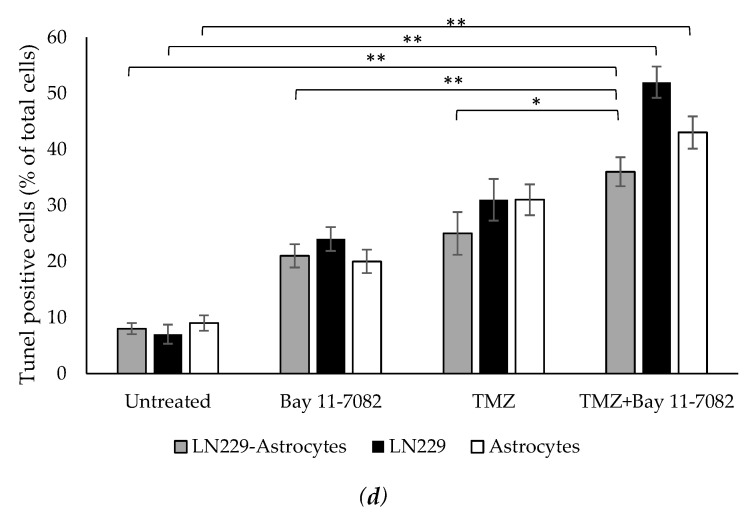
Fluorescent images of TUNEL (+) cells (green) in co-cultured and mono-cultured cells. (**a**) LN229–astrocytes co-culture. (**b**) LN229 mono-culture. (**c**) Astrocytes mono-culture. TUNEL assay was performed on cells treated with TMZ and/or Bay 11-7082 in the microwells. Cells were collected from the microwells, trypsinized, and re-plated into 8-well chamber slides. Nuclei were counterstained with DAPI (blue). (**d**) Numbers of TUNEL (+) cells are presented as mean ± SD of three biological replicates. * indicates *p* < 0.05, ** indicates *p* < 0.01. X20 objective. Scale bars, 100 µm.

In addition to cell viability assay, we further investigated how drugs affect the cell apoptosis. Therefore, we performed TUNEL assay. Co-cultured and mono-cultured cells were treated with or without TMZ and Bay 11-7082 and subjected to TUNEL assay to detect DNA damage (Figure 3a–c). The results showed that TUNEL (+) cells in the co-treatment in the co-culture were increased 4.5-fold compared with the untreated and 1.71- and 1.44-fold compared with TMZ alone and Bay 11-7082 alone, respectively (*p* < 0.01) (*p* < 0.05) (Figure 3d). TUNEL (+) cells in the co-treatment in the LN229 mono-culture were increased 7.42-fold compared with the untreated (*p* < 0.01) and 2.16- and 1.67-fold compared with TMZ alone and Bay 11-7082 alone (Figure 3d). TUNEL (+) cells in the co-treatment in the astrocytes mono-culture were increased 4.77-fold compared with the untreated (*p* < 0.01) and 2.15- and 1.38-fold compared with TMZ alone and Bay 11-7082 alone (Figure 3c).

### 2.3. Gene Expression Studies

**Figure 4:**
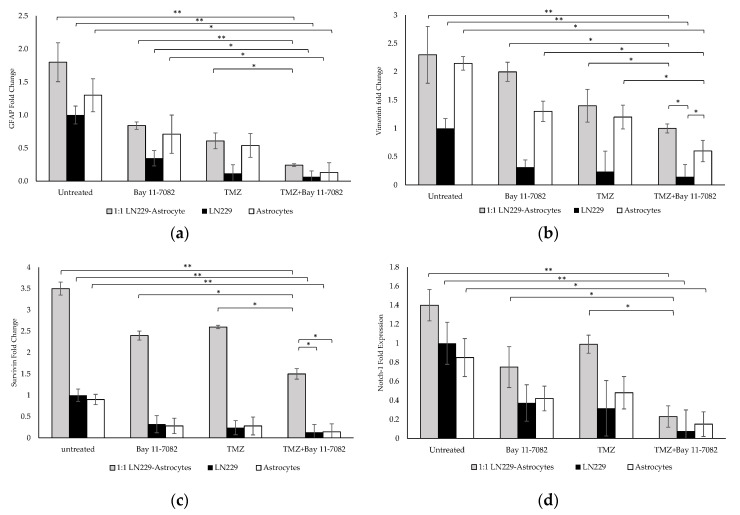
Gene expression analysis. Fold changes of (**a**) glial fibrillary acidic protein (GFAP), (**b**) vimentin, (**c**) Notch-1, and (**d**) survivin genes in the co-culture and mono-cultures. LN229 cells and astrocytes were treated with or without drugs for 7 days. Results were normalized to GAPDH total RNA level. Data represent the mean ± SD of three biological replicates. * indicates *p* < 0.05, ** indicates *p* < 0.01.

The molecular characterization and the interaction of GBM cells with astrocytes were investigated after the drug treatment. Gene expression studies were carried out using the co-culture and mono-culture spheroids treated with TMZ and/or Bay 11-7082. 3D spheroids in mono-culture without drug treatment were used as a control. Our results indicated that after the combined drug treatment, gene expressions of GFAP, vimentin, Notch-1, and survivin were significantly downregulated by 0.24-, 1-, 1.5-, and 0.23-fold, respectively, in the co-culture, 0.06-, 0.14-, 0.13-, and 0.07-fold, respectively, in the LN229 mono-culture (*p* < 0.01), and 0.13, 0.6, 0.14, and 0.15 in the astrocyte mono-culture (*p* < 0.05) (Figure 4a–d).

When co-cultured cells were treated with Bay 11-7082 alone, GFAP expression was significantly changed by 0.28-fold (*p* < 0.01) and expressions of vimentin, Notch-1, and survivin genes were significantly higher (0.5-, 0.62-, and 0.31-fold, respectively (*p* < 0.05)), compared with the combined drug treatment. When co-cultured cells were treated with TMZ alone, the expressions of GFAP, vimentin, notch-1, and survivin were changed by 0.40-, 0.71-, 0.57-, and 0.23-fold, respectively, compared with the combined drug treatment (*p* < 0.05). When mono-cultured LN229 cells were treated with Bay 11-7082 alone and TMZ alone, the expressions of GFAP, vimentin, Notch1, survivin were changed by 0.17-, 0.45-, 0.40-, 0.18-fold and 0.22-, 0.60-, 0.56-, 0.23-fold, respectively, compared to combined drug treatment (*p* < 0.05). When mono-cultured astrocytes were treated with Bay 11-7082 alone and TMZ alone, the expressions of GFAP, vimentin, Notch-1, and survivin were changed by 0.18-, 0.46-, 0.5-, 0.35-fold and 0.24-, 0.54-, 0.51-, 0.31-fold, respectively, compared to combined drug treatment. Expressions of vimentin, Notch-1, and survivin were significantly downregulated in both co- and mono-cultures after TMZ alone compared with the combined drug treatment. These results showed that combined drug treatment had more impact on the down-regulation of the GFAP and vimentin proteins that play role in the astrogliosis as well as survivin and Notch-1 that promote the tumor malignancy in the co-culture compared with the mono-cultures. These data suggested enhanced combinatorial effect of TMZ and Bay 11-7082 on the LN229–astrocytes co-culture.

### 2.4. Western Blot

**Figure 5:**
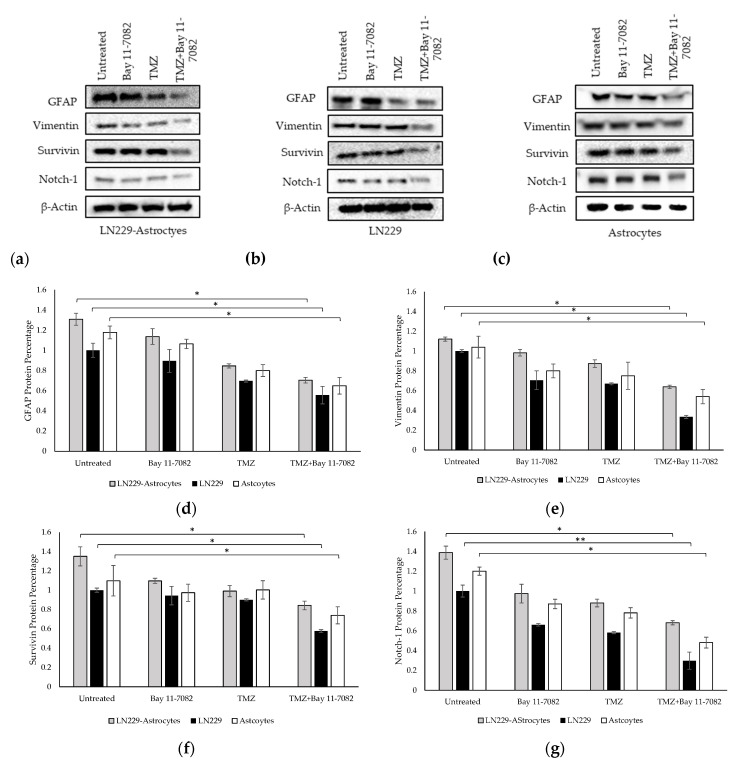
Western blot images of GFAP, vimentin, survivin, and Notch-1 in (**a**) co-culture and (**b**) LN229 mono-culture and (**c**) astrocytes mono-culture treated with or without drugs for 7 days. (**d**) Quantification of the fold change of GFAP protein. (**e**) Quantification of the fold change of vimentin protein. (**f**) Quantification of the fold change of survivin protein. (**g**) Quantification of the fold change of Notch-1 protein. The levels of the proteins were quantified using ImageJ. Data were normalized to β-Actin. Data represent the mean ± SD of three biological replicates. * indicates *p* < 0.05, ** indicates *p* < 0.01.

We performed Western blotting to analyze the expressions of GFAP, vimentin, Notch-1, and survivin proteins in the TMZ and/or Bay 11-7082 treated co-cultured and mono-cultured cells (Figure 5a–g). 3D spheroids in the mono-culture without drug treatment were used as the control group and all protein levels were normalized to β-actin housekeeping gene.

The band intensities of GFAP, vimentin, Notch-1, and survivin proteins after combined drug treatment were determined as 0.70, 0.63, 0.68, and 0.84, respectively, in the co-culture and, 1.30, 1.12, 1.38, 1.34, respectively, in the untreated group of the co-cultured cells. These results showed that after combined drug treatment, GFAP, vimentin, Notch-1, and survivin expressions in the co-culture were significantly downregulated compared with untreated LN229 mono-culture group (*p* < 0.05). In the LN229 mono-culture, the band intensities of GFAP, vimentin, Notch-1, and survivin proteins after combined drug treatment were 0.55, 0.33, 0.29, and 0.57, respectively. In the astrocyte mono-culture, the band intensities of GFAP, vimentin, Notch-1, and survivin proteins after combined drug treatment were 0.91, 0.86, 0.74 and 0.88, respectively. The results showed that after combined drug treatment, GFAP, vimentin, survivin expressions (*p* < 0.05), and Notch-1 (*p* < 0.01) were significantly downregulated compared with the mono-cultured untreated groups.

## 3. Discussion

The current standard of care for GBM is maximum surgical resection combined with radiation and TMZ therapy. However, TMZ resistance is responsible for GBM tumor recurrences in most patients [33]. Therefore, there have been several attempts using combined drug treatment to develop new strategies for GBM treatment. Among the signaling pathways in GBM, NF-κB activation play an important role in promoting tumor pathobiology and response to therapy [42,43]. NF-κB is a multi-subunit transcription factor consisting of p50 (NF-κB1, p105), p52 (NF-κB2, p100), p65 (relA), and relB. These proteins exist as homo- and hetero-dimers with the most abundant form being p50/p65, and they are activated by multiple interrelated pathways. Some studies have also reported their expression in activated microglia [44]. Astrocytes are the most diverse glial cells in the CNS and are responsible for promoting GBM development. Interaction and communication between astrocytes and GBM cells via gap junction leads to increased intercellular calcium and resistance to chemotherapy [42]. Several pathways are involved in the interaction of GBM with astrocytes, such as NF-κB signaling pathway which regulates astrocyte formation in GBM tumor microenvironment [45]. Although several studies have shown that NF-κB signaling in astrocytes can contribute to pro-inflammatory responses following injury [46], there is still a lack of understanding of the effect of NF-κB on GBM tumor microenvironment and its interaction with TMZ.

A poor clinical outcome of chemotherapy can be attributed to the usage of 2D monolayer cultures as the initial drug screening method that frequently produces inaccurate results and does not predict chemoresistance [47]. 3D tumor models have the ability to mimic tumor microenvironment and provide more accurate toxicity information for modeling drug and radiation responses [48,49]. It has been shown that cells cultured in the 3D model show significant effects on clonogenic survival in patient-derived GBM [49] and were more resistant to TMZ compared to the 2D model, and this resistance was potentiated by hypoxia [50]. These data restated the similarity of 3D cultures to in vivo TMZ resistance. To understand the impact of astrocytes on GBM cell response to drug treatment, we established a 3D co-culture model using LN229 cells and astrocytes. In addition, to assess whether the astrocytic activation was due to GBM cells, we cultured astrocytes and LN229 alone in the microwells. We assessed the response of these co-cultured and mono-cultured cells to two different drugs, TMZ and NF-κB inhibitor Bay 11-7082. The cell viability assay showed a decrease in both co-cultured and mono-cultured cells on day 7 after TMZ and/or Bay 11-7082 administration. However, the co-cultured cells were more resistant to the combined drug treatment compared to both mono-cultures. The size of the spheroids showed a similar pattern, which showed bigger spheroids in the co-culture after combined drug treatment compared to both mono-cultured cells. We also performed TUNEL apoptosis assay to support our cell viability assay. These data showed significantly less apoptotic cells in the co-culture after combined drug treatment compared to both mono-cultures and more dead cells after combined drug treatment in the LN229 mono-culture compared to the co-cultured cells. Thus, we concluded that the combined drug treatment was more effective on the cells compared with the single drug administration, and the reactive astrocytes in the co-culture might have increased the drug sensitivity of the GBM cells to both drugs.

Reactive astrocytes protect GBM cells from TMZ and Bay 11- 7082-induced apoptosis and have been shown to suppress the expression of MGMT in TMZ resistant GBM cell lines [8]. Therefore, we hypothesized that TMZ–Bay 11-7082 treatment can reduce astrocyte transformation and thus affect the expression of the proteins playing role in the tumor development and malignancy in the GBM–astrocyte co-culture. We investigated the expressions of several important markers in GBM development. GFAP is one of the most common markers in astrocyte differentiation and GBM development. Our findings first showed that GFAP was expressed in both co-culture and mono-culture. Vimentin, along with GFAP, is present in mature astrocytes as well as in LN229 cells [51]. Although vimentin is the major cytoskeletal component present in immature glia, it is expressed to the same extent in all grades of astrocytic tumors and correlated with the malignancy grade in gliomas [52]. Our qPCR showed that the expressions of both GFAP and vimentin genes in the co-culture were higher compared with the mono-culture for all treatment groups. This result confirmed the presence of reactive astrocytes in our 3D co-culture model. When we applied combined drug treatment, the expression of both GFAP and vimentin significantly decreased compared with both single drug treatment groups. This result implied that when TMZ was applied with Bay 11-7082, it showed more effect on the signaling pathways of GBM tumor than the single drug treatment. Western blot results confirmed that the downregulation in these genes was translated into protein expression. We observed significantly decreased expressions of GFAP and vimentin in the co-culture after TMZ- Bay 11-7082 treatment compared with the single drug treatment. The expression of these two markers pointed towards astrocytic lineage in the GBM cells [19]. Altogether, these results might imply that reactive astrocytes promoted GBM cells viability in the co-culture [53].

This astrocytic protection could be via gap junction communication (GJC) and the upregulation in the expression levels of malignancy genes such as Notch-1 and survivin [54]. Compared with the GBM mono-culture, reactive astrocytes promoted the upregulation of GFAP and vimentin proteins in the tumor microenvironment and possibly formed a functional barrier called a glial scar, thus increasing GBM chemoresistance [55], that needs to be further investigated. Notch-1 signaling is an evolutionarily conserved pathway that regulates cellular interactions and processes. Previous studies have shown that Notch-1 is upregulated in many glioma cell lines and primary human gliomas by promoting cell survival, proliferation, and invasion [56,57]. It has also been shown that several pathways including NF-κB can promote Notch-1 signaling, where NF-κB subunit p65 binds to the Notch-1 promoter and activates it [58]. Considering the importance of Notch-1 signaling in glioblastoma development, Notch-1-antagonizing strategies may hold great promise for therapeutic approaches. Although reactive astrocytes potentially increased the resistance of the co-culture to the drug treatment, our GBM–astrocyte co-culture treated with the combination of TMZ- Bay 11-7082 showed a significant decrease in both Notch-1 gene and protein expressions compared to the untreated group as well as the single drug treatments. These data suggest that the combined drug treatment could target Notch-1 signaling and, thus, the proliferation and Notch-1-dependent apoptosis-regulating pathway characterized in GBM [56]. Survivin is a member of the inhibitor-of-apoptosis (IAP) family [57]. Survivin expression has been found to be undetectable in normal adult tissues [59]. However, its expression has been reported in different tumor types including colorectal, breast, and ovarian [60,61]. Its prognostic value has also been investigated in gliomas [62]. Survivin expression in astrocytic tumors has been shown to vary with tumor malignancy [22,63]. However, its interaction with the tumor progression and resistance to drugs remain unclear. NF-κB, which is considered as a pro-survival factor, promotes cell survival and proliferation. Proteins induced by NF-κB in GBM that act in this manner include Bcl-2, Bcl-xl, and the inhibitor of apoptosis proteins (IAPs) such as survivin. We have already shown in our lab that Bay 11-7082 in combination with TMZ modulated anti-apoptotic protein Bcl-2 and pro-apoptotic protein Bax in the NF-κB signaling pathway in 3D patient-derived GBM cells [64]. In this study, we further investigated the effect of the combined drug treatment on the survivin expression. As we discussed in the previous paragraphs, reactive astrocytes increased the resistance of the co-culture to the drug treatment compared with mono-cultures, and survivin expression was higher in the co-culture than the mono-cultures. However, we observed a significant decrease in the survivin gene expression compared with the untreated group as well as the single drug treatments. This result suggested that survivin expression was positively and significantly associated with GBM proliferation and susceptibility to drugs.

As a summary, in this study, we investigated the efficacy of 3D LN229 GBM cells–astrocyte co-culture model to mimic the tumor microenvironment and modulate the sensitivity of GBM cells to combined TMZ and NF-κB inhibitor Bay 11-7082 treatment. Our results suggested that TMZ in combination with NF-κB inhibitor Bay 11-7082 increased GBM survival and resistance. However, considering that LN229 is a commercial non-stem GBM cell line [65,66], future studies should investigate the sensitivity of the astrocyte co-culture model with other GBM cell lines, including T98G, U251, U87 [66] and patient-derived primary GBM cell lines with or without stem cells [67], to TMZ in combination with NF-κB inhibitor Bay 11-7082 or different drug combinations.

## 4. Materials and Methods

### 4.1. Cell Lines and Cell Culture

Glioblastoma LN229 cell line was purchased from the American Tissue Culture Collection (ATCC, Manassas, VA, USA). LN229 cells were cultured in Dulbecco’s modified Eagle’s medium (DMEM) (Corning, New York, NY, USA) supplemented with 10% (*v*/*v*) fetal bovine serum (FBS) (VWR, Dallas, TX, USA) and 1% (*v*/*v*) penicillin/streptomycin (VWR, Dallas, TX, USA). Primary human astrocytes were obtained from ScienCell (Carlsbad, CA, USA). They were cultured in the basal medium supplemented with 2% (*v*/*v*) fetal bovine serum, 1% (*v*/*v*) astrocyte growth supplement, and penicillin/streptomycin. All cells were maintained under sterile tissue culture hoods and kept in a 95% air-5% CO2 humidified incubator at 37 °C.

### 4.2. Microwell Fabrication

Microwell fabrication process was followed as described previously [33]. Briefly, 25 × 25 mm cover glass slides were washed and treated with 3-(Trimethoxysilyl) propyl methacrylate 98% (TMSPMA) (Life Technologies, New York, NY, USA). Then, the treated slide was covered with 20 µL of 40% (*w*/*w*) PEGDA (MW 700) (Life Technologies, New York, NY, USA) solution containing 0.2% (*w*/*v*) of the photoinitiator (PI) 2-hydroxy-2-methyl propiophenone dissolved in Phosphate Buffered Saline (PBS) (Life Technologies, New York, NY, USA). The glass slide was exposed to Lumen Dynamics the OmniCure^®^ Series 2000 (Lumen Dynamics Group Inc, Mississauga, ON, Canada) for 30 s at a working distance of 6 inches. At the end, 300 µL of prepared PEGDA solution was added to the slide and exposed to UV light for 36 s with the desired photomask on top of it. The photomask was purchased from (CADart, Bandon, OR, USA) and designed in round pattern of 400 µm in diameter with AutoCAD (Autodesk Inc, San Rafael, CA, USA).

### 4.3. Co-Culture of Glioblastoma Cells and Astrocyte

LN229 cells and astrocytes were first cultured in the cell culture plates until they reached their confluency. Then, they were trypsinized and plated in microwells with a density of 0.2 × 10^6^ cells/mL and a ratio of 1:1 (GBM:Astrocytes) and cultured in the astrocyte medium. Astrocytes used for this experiment were between passages 2 and 4. The same number of the cells were used for both mono-culture and co-culture. LN229 cells were stained with 10 µM of CellTracker Red CMTPX fluorescence dye (Thermo Fisher Scientific, Houston, TX, USA) and astrocytes were stained with 10 µM of CellTracker Green CMFDA fluorescence dye (Thermo Fisher Scientific, Houston, TX, USA) in order to distinguish between two cell lines. LN229 cells and astrocytes were mono-cultured with a density of 0.2 × 10^6^ cells/mL and kept in the same culture condition. Cell spheroid formation inside of the microwell was monitored using an Olympus fluorescence microscope (Olympus, Tokyo, Japan)

### 4.4. Drug Administration

After 7 days of spheroid culture in the microwells, TMZ and/or Bay 11-7082 were introduced to the cells in the microwells. TMZ (Thermo Fisher Scientific, Houston, TX, USA) was dissolved in dimethylsulfoxide (DMSO) (Santa Cruz Biotechnology, Dallas, TX, USA) to prepare a solution of 10 mM TMZ. The solution was diluted to 600 µM TMZ in astrocyte media. Bay 11-7082 was dissolved in DMSO and diluted to 10 µM using appropriate media [11]. Cell culture media was removed from the microwells and replaced with 600 µM TMZ and/or 10 µM Bay 11-7082 for the co-culture and mono-cultures. Then, the spheroids were cultured in the microwells for 7 more days. Untreated microwells were maintained under the same conditions.

### 4.5. In Situ Apoptosis Assay (TUNEL)

LN229 cells and astrocytes, co- or mono-cultured in the microwells, were treated with TMZ and/or Bay 11-7082 for 7 days. They were collected from the microwells and trypsinized to assess DNA fragmentation with TACS^®^ 2 TdT-Fluor In Situ Apoptosis Detection Kit (R&D Systems Inc., Minneapolis, MN, USA) following the manufacturer’s instructions. Labelled cells were examined using fluorescence microscopy (Olympus, Tokyo, Japan) using a 405∼488 nm filter [64]

### 4.6. Gene Expression Analysis by Quantitative PCR (qPCR)

Total RNAs from LN229 cells and astrocytes were extracted using RNeasy Mini Kit (Qiagen, Germantown, MD, USA) according to the manufacturer’s instructions. Nanodrop (2000 series) (Thermo Fisher Scientific, Houston, TX, USA) was used for qualification and quantification of the RNA by reading the optical density (OD) at 260 and 280 nm. Then cDNA was synthesized from RNA samples using High-Capacity cDNA Reverse Transcription Kit with RNase Inhibitor (Thermo Fisher Scientific, Houston, TX, USA) by following manufacturer’s protocol. Quantitative PCR experiments were performed using StepOnePlus Real-Time PCR System (Thermo Fisher Scientific, Houston, TX, USA). Briefly, a reaction mixture of 20 µL, containing 10 µL of PerfeCTa SYBR Green SuperMix Reaction Mixes (Quanta bio, Beverly, MA, USA), 300 nM primers, and 50 ng cDNA. Then, qPCR instrument’s thermal cycling was programmed for 40 cycles at 95 °C for 15 s and 53 °C for 45 s after initial incubations at 95 °C for 10 min. GFAP, vimentin, Notch-1, and survivin expression was investigated with their own primer sequences of tested genes listed in Table 1 [25,60,61]. Target gene expression was normalized to GAPDH [36] levels in the same reaction using the ΔΔCt method.

### 4.7. Western Blot

LN229 and astrocytes were co- or mono-cultured in the microwells, treated with TMZ and/or Bay 11-7082, and collected from the microwells. Then, they were washed with PBS protein, isolated, and lysed using RIPA lysis buffer (20-mM HEPES, pH 7.0, 200-mM NaCl, 1-mM EDTA, 1-mM EGTA, 1% Triton X-100, 5-mM sodium pyrophosphate, 80-mM β-glycerophosphate, 50-mM NaF, 0.1% SS) including freshly added protease inhibitor cocktail (Sigma-Aldrich, St. Louis, MO, USA) and phosphatase inhibitor cocktail 3 (Sigma-Aldrich, St. Louis, MO, USA). Cell lysates were incubated on ice for 30 min and centrifuged at 4 °C, 14,000 rpm for 10 min. Supernatants were collected and the concentration of protein was measured using Bradford Protein reagent (Bio-Rad, Hercules, CA, USA). Equal amounts of cell lysates were subjected to 4%–20% SDS-PAGE gels, transferred to a PVDF membrane (Thermo Fisher Scientific, Houston, TX, USA). Membranes were blocked with 5% milk (in 1× PBS-Tween20) for 30 min, followed by primary antibody incubation overnight at 4 °C. After three washes with PBS-Tween20 (5 min each), membranes were incubated with secondary antibody (5% milk in PBS-Tween20) for 1 h. Protein bands were visualized using ECL Western blot detection system (Amersham Pharmacia Biotech). The data were normalized to β-actin. GFAP, vimentin (Santa Cruz, Dallas, TX, USA) Notch-1, β-Actin (Abcam, Cambridge, MA, USA), and survivin (Cell Signaling Technology, Danvers, MA, USA) antibodies were used at a concentration of 1:1000. Goat anti-Mouse IgG (H+L) Secondary Antibody (HRP) (Novus Biologicals, Centennial, CO, USA) was used at 1:2000 concentration.

### 4.8. Statistical Analysis

All results were from three independent experiments performed in triplicate. Statistical analysis was calculated using Student’s t-test. Confidence intervals were set at 95% (*p* < 0.05) and 99% (*p* < 0.01). Error bars are mean ± standard error.

## Figures and Tables

**Table 1:** Primer sequences used in quantitative polymerase chain reaction (qPCR).

Gene	Forward Primer	Reverse Primer
GFAP	CCAAACTGGCTGACGTTTACC	TGGTTTCATCTTGGAGCTTCTG
Vimentin	CCC TCC GCA GCC ATG	ATG AGT GCT GCA CTG AGT GTG
Notch-1	ATTTCACCGTGGGTGC	GTGTATCGGGCCCATCATGC
Survivin	CCGGGATCCATGGGTGCCCCGACGTTG	CGCGAATTCAGAGGCCTCAATCCATGG
GAPDH	CTCTGCTCCTCCTGTTCGAC	AAATGAGCCCCAGCCTTCTC

## Abbreviations

GBM	Glioblastoma multiforme
TMZ	Temozolomide
3D	Three-dimensional
PEGDA	Poly (ethylene glycol) dimethyl acrylate
qPCR	Real-time polymerase chain reaction
MGMT	O^6^-methylguanine-DNA methyltransferase
GFAP	Glial fibrillary acidic protein
CNS	Central nervous system
IF	Intermediate filament
EMT	Epithelial to mesenchymal transition
ECM	Extracellular matrix
TMSPMA	3-(Trimethoxysilyl) propyl methacrylate
GJC	Gap junction communication
DMSO	Dimethylsulfoxide
